# Five Resections of the Alimentary Canal

**Published:** 1891-01

**Authors:** B. E. Hadra

**Affiliations:** Galveston


DANIEL'S

### Texas Medical Journal.

A Representative Organ of the Medical Profession, and an Exponent of Rational Medicine; devoted to the Organization, Advancement and Elevation of the Profession in Texas.

PUBLISHED MONTHLY.—SUBSCRIPTION $2.00 A YEAR.

Vol. VI. AUSTIN, JANUARY, 1891. No. 7.

### Original Articles.

CONTRIBUTED EXCLUSIVELY TO THIS JOURNAL.

*The Articles in this Department are accepted and published with the understanding that we are not responsible for, nor do we indorse the views and opinions of the writers by so doing.*

---

For Daniel's Texas Medical Journal.]

### FIVE RESECTIONS ON THE ALIMENTARY CANAL.

BY B. E. HADRA, M. D., GALVESTON.

AMONGST a larger number of intestinal operations, which I lately performed, there were five cases of resections. They form the subject of the following report:

CASE I.—GANGRENE OF BOWEL—RESECTION.

A robust farmer, some thirty years of age, living at Alvin, was suddenly seized with an incarceration of an old reducible hernia on the right side. It was reduced by taxis by a Houston surgeon, but unfortunately]"en masse," as the further progress of the case showed. Three days afterward Dr. H. A. West and myself went to see patient, and with the assistance of Dr. Fields, he was operated on, as obstruction of bowel persisted and all the signs of sepsis were present. The scrotum was mortified, black and pulpy, and inflammatory reddening with œdematous infiltration extended over the right abdominal half. The case, of course, looked so hopeless that only his wish to be operated on, and his good pulse and good spirits prevailed on us to proceed.

After the usual preparations, an oblique incision over the external inguinal ring, extending about three inches in either direction, was made, and a totally grangrenous loop of small intestine was exposed. The black necrotic piece was of about 5 ″ length and the demarkation lines well defined. It was removed with its mesentery by the usual triangular exsection, the cut ends closed with two rows of stitches (one after Czerny, the other Lembert's), and lateral anastomosis established. Next the perfectly necrotized testicle, with its covers, the hernial sac, the cord, etc., was removed, also the grangrenous, slate-colored peritoneal and fascial lining of the abdominal parietes to such an extent as seemed prudent. The wound was left open for drainage, as the extent of gangrene in the latest named tissues forbade their total removal. Duration of operation about one hour. Patient rallied well from it, but slowly became comatous, and died under symptoms of sepsis six hours after operation.

CASE II.—PISTOL WOUNDS OF ILIUM.

A colored boy, of about nineteen years, was brought to the John Sealy hospital, at 11 o'clock p. m., on the 24th of February, 1890. He was shot, shortly before, right below right margin of ribs, with a six-shooter (calibre 38). He was operated on next morning at 9 o'clock; temperature having become feverish. The wound was incised downward for four to five inches, and a loop of small intestine with five holes brought easily to light. There was already a fully established periionitis present. A large amount of bloody serum, mixed with fecal matter, poured out, and the gut was covered with fibrinous flakes. Four of the holes were near together and so arranged as to involve about half of the circumference of the gut, whilst the fifth opening was about two inches distant. It was considered safer to exsect the whole injured piece, than to sew each hole, as most likely a stricture would have resulted from the latter method. Usual triangular resection through gut and mesentery and circular union of cut-ends by two rows of suture (one Czerny, one Lembert) was made. Abdominal cavity washed out and two large drainage tubes inserted, around which the abdominal incision was closed. Fever increased steadily, a soporose condition set in a few hours after operation, from which he had rallied nicely. He died at 8 o'clock in the evening.

Post-mortem examination showed a general peritonitis, though the line of suture held very well. Bullet could not be found, but there was bruising of posterior wall of abdomen at a spot to the right of vertebral column. Very likely the bullet had lodged in the depth of the lumbar muscles.

### CASE III.—ULCERATION AND PERFORATION OF ILLIUM.

Mrs. S., a Hebrew lady, 53 years of age, consulted me for prolapse of womb. She complained of dragging pains in pelvis, leucorrhea, poor digestion, thought she had a little fever in the evening, bowels constipated but otherwise giving no trouble. She felt very much prostrated and hardly able to attend to her household duties. Examination revealed a very excessive prolapse, with excoriations of vagina and crevix. In the left side of pelvis, in a region usually called ovarian, there could be felt a nodulated tumor of the apparent size of a small fist. It was tender on pressure, seemed to be little moveable and was not in connection with womb. Could be felt and distinctly outlined by bimanual manipulation. It was clearly not uterine, as the uterus could be dragged out without changing the tumor's situation, also its connection with the bowels was improbable, neither the previous history nor the symptoms giving any evidence of it; thus it was considered to be most likely ovarian, either a sarconia or a fibroma, or a dermoid cyst. Still the diagnosis was considered dubious because of the absence of grave suffering and of hydropic signs. Besides, if it were ovarian, it would have been bound to follow the prolapsing womb to some extent, which it did not do. Dr. A. Fly concurred in my opinion.

Operation on the 25th of July, 1890, Drs. Dock, Fly, Hadra, jr., and Sykes assisting. After the usual incision in middle line and the insertion of hand into cavity, fetid pus escaped at once. An abscess cavity was found constituted by the walls of two contiguous loops of bowel and their mesentery. The inside of the abscess cavity was of black color, ragged surface and communicated with the gut by an opening admitting the tip of a finger. Around this perforation the gut was thickened, reddish and soft, resembling medullary carcinoma. The question now arose whether the hole should be sewn up only, or whether the gut should be stitched into the parietal incision (establishment of an artificial anus), or whether the diseased portion removed. We decided to do the last named operation, as otherwise diseased parts would have been left behind, which would, no doubt, have led to similar trouble in the near future. Besides, it could not be made out on the spot whether the new formation was malignant or not. A triangular resection of a piece of small intestine of about five inches in length with its mesentery, was made, and the cut ends brought together by a double row of sutures. Adhesions between neighboring folds of intestine were not interfered with. Drainage, etc. Operation lasted over an hour. Patient rallied well, but further progress was not so satisfactory. Persistent nausea, high septic fever, no rest except by morphlne, and all the annoyances of slow peritonitis followed. On examination per vaginum a bogginess in left parametrium was detected, and two weeks after operation reopening of the not yet perfectly closed wound (track of drainage tube) and washing out of cavity was decided on. Of course, an accumulation of inflammatory material in the depths of pelvis was expected.

When we arrived at the house we were informed that shortly before a large quantity of pus had passed by the rectum. Perforation had no doubt occurred, still, although most likely the fullness felt by me, had something to do with this new abscess, reopening was considered proper, and was done. Douglas' pouch was then well washed out, but the left parametrium, where indeed an intestinal loop was found adherent to pelvis, and which, no doubt contained the perforation also, was left alone, trusting to the drainage per rectum, and fearing new injuries to the diseased gut deep down in the pelvis, which may have necessitated a new resection. Next day a large swelling, or rather a tumor, made its appearance right over the bend of the ascending colon; it was taken for adherent omentum, treated with warm application and disappeared after some time.

Patient, after all this, had a very hard time for several weeks, a constant retching and nausea and a most peculiar burning in æsophagus and stomach. The discharge by the rectum ceased in a few days. Patient made a slow but perfect recovery, and could be pronounced well about ten weeks after first operation. The prolapse of uterus also improved somewhat, most likely from new adhesions caused by pelvic peritonitis.

Dr. G. Dock's examination of excised part gave the following results: Excised piece about five inches in length, most probable from middle of ilium. The internal wall is thickened and injected, the serosa roughened. Mesentery ¼ to ½ inch thick and shows intense and extensive infiltration of small cells between and beneath the tubules and in the submocosa. This is most marked near the opening where there is extensive hemorrhage beneath the mucosa. Sections of the mesentery show a great subserous hemorrhage and small celled infiltration. The ulcerated part shows a thin necrotic layer. In other words: There is hemorrhage and purulent inflammation of the intestine, the condition of the mesentery corresponding. It is highly probable the latter condition is the result of a sloughing gland, but the relation of one to the other is not clear. Most frequently in such cases the lymph gland is primarily affected (tuberculosis usaally) and perforated the gut. There is here, however, no trace of tuberculosis. Circumscribed inflammation does occur in the bowels, and this may be such a case, but the exciting cause cannot be discerned in the tissues. Unfortunately the pus which escaped at the operation, was not examined microscopically; to the naked eye it presented nothing peculiar.

The pus which was passed per rectum at a later period showed no organism except small bacteria and cocci.

CASE IV.—PYLORECTOMY FOR CANCER.

Mrs. S., a German farmer's wife, 24 years old, of good family history, had suffered for two years from gastralgia, vomiting, occasionally of blood, emaciation, etc. She was treated for several affections ot stomach. In December, 1889, a tumor was detected in abdomen and taken for a movable kidney. Symptoms grew worse and worse, especially when pregnancy set in again. Four weeks after a normal delivery at term she was examined by me, and a distinct tumor of the size of a hen's egg easily mapped out to the right of the middle line, about five inches below margin of ribs. It was nodulated, painful on pressure, and movable in every direction to a distance of one to two inches. Patient did not retain anything, threw up every meal and was extremely emaciated. Strange to say, she nursed her babe, at least, she attempted to do so, assisted by the bottle.

Operation was proposed and accepted as the only chance, the diagnosis being plain enough. She was fed by rectal injections and taken to John Sealy hospital three days before operation, which, after due preparation, washing of stomach, bathing, etc., was performed on the 28th of October, 1890.

Chloroforms-narcosis, median incision. The rules worked out by Billroth were strictly followed. The incision, after detaching the omental connections, was started on the small curvature, where the new growth extended over half of the extremely small stomach; it was then carried obliquely toward the pylorus to a point where the future pyloric outlet would likely have to be created. This wound was first stitched up, before progressing, in order to avoid too great a loss of blood. Three rows of suture were used; one through the mucous membrane, one after Czerny, one after Lembert. The further excisions were made vertically through the remaining portion of stomach and duodenum, and as the circumferences of the two openings fitted well together, they were united by a row of sutures, through the mucous membranes, as much as it was feasible, and then again from the outside by a Czerny and a Lembert row, which latter had to gap posteriorly, owing to the absence of peritoneum there. The omental wounds were oozing somewhat, and I thought best to leave an iodoformed gauze-strip, folded upon itself, in the two pockets, formed above and below the new pylonic portion, and also surrounding this latter in front. The other end of it was carried through the abdominal incision, which then was closed up around it. (This wick was left in for five days when it was removed with ease.) Nothing of importance occurred in the next few days. A little nausea occasionally, and only a few times temperature over 100. On the fourth day milk was taken by the mouth. The only trouble was given by the track of the gauze, which closed up very slowly.

Patient gained steadily, had no pain whatever, but still now there is an occasional vomiting whenever she is imprudent in her diet. The stomach can hardly be larger than a big fist, unless it has again become distended, and when overladen vomiting is but natural. Her bowels were not acting regularly in the beginning but do so now.

Dr. Dock's pathological report: *Cancer of stomach.* The diseased part extends from just within the pylonic ring to a distance of 3½ inches toward the cardia. It is most extensive on the lesser curvature, though the degeneration surrounds the pylonic end of the stomach entirely. The mucous membrane is thickened and thrown into folds and of a bright pink color. Running along the lesser curvature is an ulcer a little more than an inch long and half an inch wide. The floor is hollowed out and slightly includes the muscularis; its walls are dense and thick, sections showing that most of the thickness is due to infiltrated submucosa, There are a few smaller ulcers, the largest of which is half an inch in diameter and extends to the muscularis. A section through the lesser curvature shows the muscularis to be hypertrophied, being about ¼ inch thick, the bundles showing plainly. At a point about two inches from the pylorus, in the lesser curvature, there is an infiltration of the subserosa. Microscopic examination shows the growth to be a medullary carcinoma. The best studies can be made through nodules not yet ulcerated. These show an excessive proliferation of the epithelium of the tubules, in which glandular structure is lost. These areas are bordered by dense small-celled infiltration from the surrounding mucous membrane. Below in some places the muscularis mucosæ can be seen perforated at intervals by strands of epithelial cells. In some places all traces of the muscularis mucosæ are gone, or a few bundles of muscular fibres can be seen lying irregularly among the cells of the new growth which penetrate into the submucosa. In such places the submucosa is transformed into a dense granulation tissue, in which glandular cell nests can only rarely be found. In some parts large masses of small epithelial cells can be seen between the bundles of the muscularis. In the part of the lesser curvature, mentioned before, a large collection of epithelial cells between muscularis and serosa, which can be traced into the lymph vessels. The epithelial elements show atrophy, fatty degeneration, and at times two or three can be seen together, which seem to be colloid. The lymphatic glands in the lesser omentum are slightly swollen, but show no signs of cancer, except one, in which however it cannot be stated with certainty, a few epitheloid cells in a sinus having a suspicious grouping.

I may add that this case is, as much as I can judge from the literature on hand, the youngest operated on for intestinal cancer. Patient was twenty-four years old and had for fully two years clear symptoms of the trouble. Whether she is permanently cured or not, I am, of course, unable to say.

CASE V.—RESECTION OF APPENDIX VERMIFORMIS FOR CHRONIC RELAPSING SO-CALLED PERITYPHLITIS.

This case is reported in the *N. Y. Record* of March 8, 1890, and I will, therefore, be short. The patient, a man of some 40 years, suffered from frequent attacks of perityphlitis to such a degree as to render his life miserable. He was operated on in a free interval, or rather right after an attack.

The appendix was found somewhat thicker than normal, and was turned forward so as to lay in front and across the cœcum. The appendix was removed and nothing found but a thickening of the walls and a catarrhal irritation of the mucous membrane. I am of the opinion that the abnormal position of the appendix, congenital or acquired, caused the trouble. Evidently every time that the cœcum became extraordinarily filled, it had to lift the appendix up and thus had to pull on the latter's mesentery. Thus the constipation and cœcal accumulation seems to me to have been the primary trouble and the appendicitis, or whatever it was, the consequence of it. The patient is, since the operation (over one year), relieved of his suffering, though constipation is still a frequent complaint. This case is the fourth reported resection of the appendix for a chronic complaint and performed in a free interval.

